# Nuclear Receptor and Stress Response Pathways Associated with Antineoplastic Agent-Induced Diarrhea

**DOI:** 10.3390/ijms232012407

**Published:** 2022-10-17

**Authors:** Mashiro Okunaka, Daisuke Kano, Yoshihiro Uesawa

**Affiliations:** 1 Department of Medical Molecular Informatics, Meiji Pharmaceutical University, Kiyose 204-8588, Japan; 2 Department of Pharmacy, National Cancer Center Hospital East, Kashiwa 277-8577, Japan

**Keywords:** drug-induced diarrhea, cancer, molecular initiating event

## Abstract

In severe cases, antineoplastic agent-induced diarrhea may be life-threatening; therefore, it is necessary to determine the mechanism of toxicity and identify the optimal management. The mechanism of antineoplastic agent-induced diarrhea is still unclear but is often considered to be multifactorial. The aim of this study was to determine the molecular initiating event (MIE), which is the initial interaction between molecules and biomolecules or biosystems, and to evaluate the MIE specific to antineoplastic agents that induce diarrhea. We detected diarrhea-inducing drug signals based on adjusted odds ratios using the Food and Drug Administration Adverse Event Reporting System. We then used the quantitative structure-activity relationship platform of Toxicity Predictor to identify potential MIEs that are specific to diarrhea-inducing antineoplastic agents. We found that progesterone receptor antagonists were potential MIEs associated with diarrhea. The findings of this study may help improve the prediction and management of antineoplastic agent-induced diarrhea.

## 1. Introduction

Diarrhea accounts for about 7% of all adverse drug events (ADEs) [1]. Many antineoplastic agents target rapidly dividing cells, so the effects on such cells in the epithelium of the gastrointestinal tract can lead to various gastrointestinal symptoms. Grade 3–4 severity diarrhea has been reported at a frequency of 5–47% in clinical trials [2,3,4,5,6,7]. With the recent introduction of tyrosine kinase inhibitors and epidermal growth factor receptor (EGFR) inhibitors, a high frequency of clinically important diarrhea has been reported [8,9]. Since antineoplastic agent-induced diarrhea may lead to hospitalization and may be life-threatening in severe cases, it is necessary to determine its mechanism and identify optimal management strategies.

Pathophysiological mechanisms of drug-induced diarrhea have been reported to include osmotic diarrhea, secretory diarrhea, short transit time, exudative diarrhea and protein-losing enteropathy, malabsorption of fats and carbohydrates, and dyspepsia. The mechanism of drug-induced diarrhea is often considered to be multifactorial but unclear [10].

Adverse outcome pathways (AOPs) [11] provide a framework for understanding the relationship between toxicological insights and meaningful endpoints of pathophysiological mechanisms. AOPs represent the sequence of events from chemical receptor activation to in vivo adverse outcome (AO) via a series of key events at the cellular and subcellular levels.

Molecular initiating events (MIEs) are generally regarded as the initial interaction between a molecule and a biomolecule or biosystem that can be causally linked to an outcome via AOPs [12]. MIEs are a concept that arose from the need to understand the chemical and biological mechanisms behind the toxicological endpoint triggered by substances. MIE targets include nuclear receptor (NR) and stress response (SR) pathways. To explore MIEs associated with antineoplastic agent-induced diarrhea, we investigated the NR and SR pathways targeted by the Tox21 program, which performs in vitro quantitative high-throughput screening of approximately 10,000 compounds. A clear understanding of MIE chemistry is essential for developing a quantitative structure-activity relationship (QSAR) and can facilitate AO predictions based on MIE activity [13]. Thus, understanding MIEs is a practical necessity in modern toxicology.

The aim of this study was to evaluate potential MIEs that are specific to diarrhea-inducing antineoplastic agents and to understand the relationship between antineoplastic molecules and pathophysiological mechanisms of diarrhea.

## 2. Results

### 2.1. Presentation of Data

**Figure 1:**
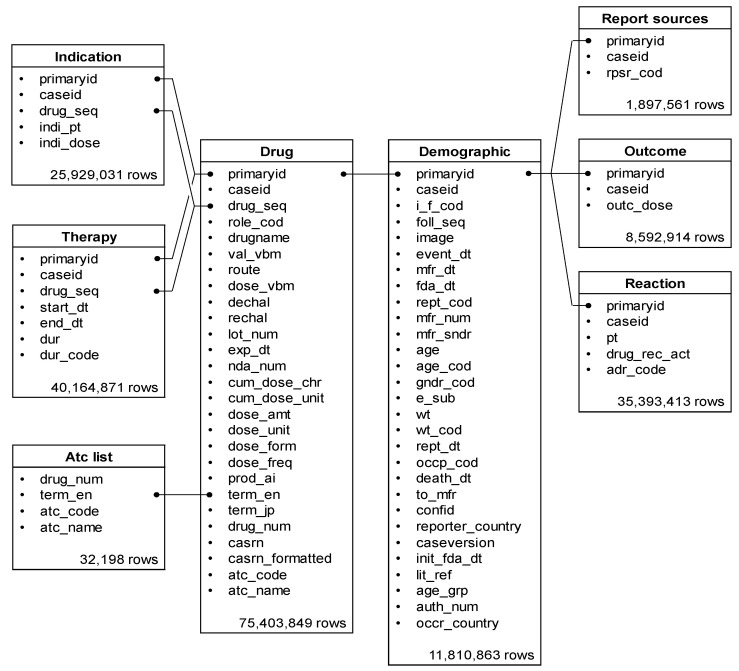
Eight information tables from the United States Food and Drug Administration Adverse Event Reporting System (FAERS) database. The number of rows equals the number of reports obtained between April 2004 and September 2020.

**Figure 2:**
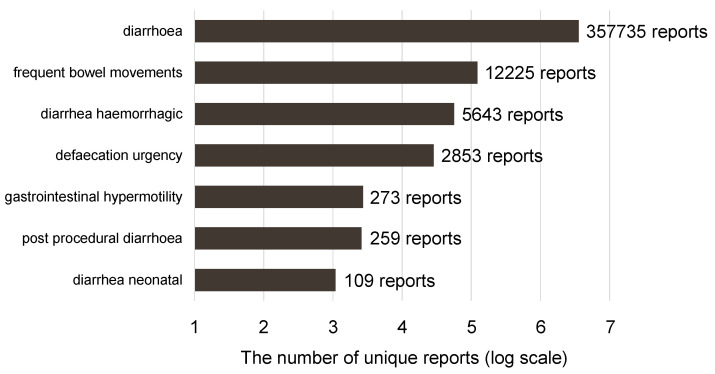
The number of unique records with preferred terms related to diarrhea, according to the narrow scope of Standardized MeDRA Queries.

Of the 35,393,413 rows (21,349 categories) of ADEs registered in the FAERS database, 379,097 rows (seven categories) were reported as diarrhea. DRUG included 75,403,849 rows, REAC included 35,393,413 rows, and the Anatomical Therapeutic Chemical list included 32,198 rows (Figure 1). The number of unique records with PTs related to diarrhea, according to the narrow scope of SMQ, is shown in Figure 2. The most common PTs in the records was diarrhea (94%, 357,735 rows).

### 2.2. Diarrhea-Inducing Antineoplastic Agents

**Figure 3:**
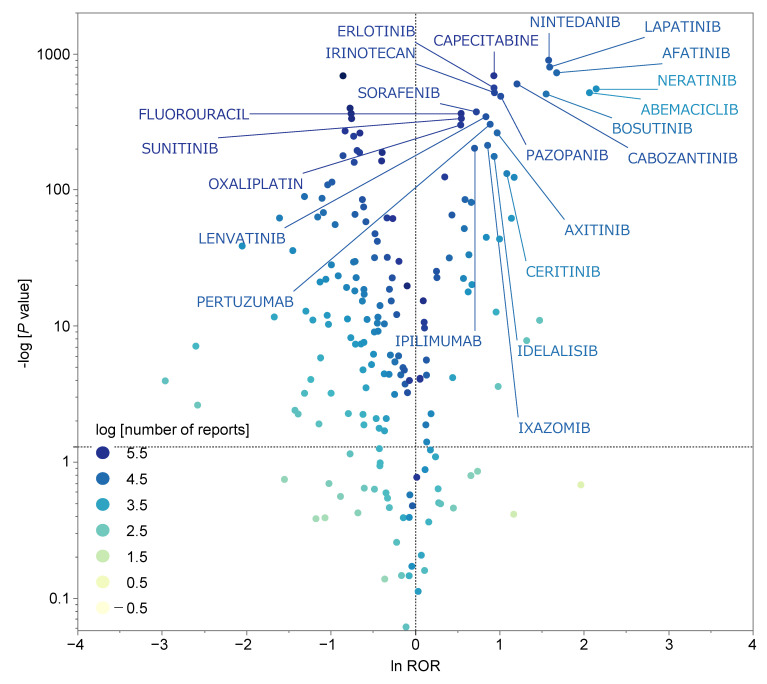
Drugs associated with the development of diarrhea. The *x*-axis shows the natural logaithms of the odds ratios (lnROR), and the *y*-axis shows the common logarithm of the inverse *p*-value (−log10[*p*-value]) from Fisher’s exact test. The ORs were calculated using cross-tabulation (Table 2). The dotted line on the *y*-axis represents *p* = 0.05. The plot colors show the common logarithm of the total number of reported adverse events for each drug.

Figure 3 shows the volcano plot of examined drugs suspected of causing diarrhea and other drugs. Drugs located on the positive side of the *x*-axis (on which InROR is plotted) were reported to cause diarrhea more than any other ADE. Drugs with high positive values on the *y*-axis (on which −log10[*p*-value] is plotted) showed great significant differences. In other words, based on ROR and statistically significant difference, the antineoplastic agents most likely to induce diarrhea appeared on the upper right side of the volcano plot.

Of the 190 drugs reported as (*a* + *c*) > 200, 55 drugs (28.9%) had lnROR > 0 and −log10[*p*-value] ≥ 1.3 (Figure 3). The 10 drugs with the highest ROR and most significant difference were neratinib, abemaciclib, afatinib, lapatinib, nintedanib, bosutinib, dacometinib, fedratinib, cabozantinib, and panobinostat (Table 1).

**Table 1:** The drugs with highest RORs of diarrhea.

Antineoplastic Agents	ROR (95% CI)	*p*-Value	Number of Report	ATC Code	ATC Name
Neratinib	8.56 (8.03–9.12)	<0.0001	8136	L01EH	HER2 tyrosine kinase inhibitors
Abemaciclib	7.90 (7.41–8.43)	<0.0001	8400	L01EF	CDK inhibitors
Afatinib	5.35 (5.17–5.55)	<0.0001	38,698	L01EB	EGFR tyrosine kinase inhibitors
Lapatinib	4.93 (4.78–5.08)	<0.0001	57,315	L01EH	HER2 tyrosine kinase inhibitors
Nintedanib	4.87 (4.74–5.00)	<0.0001	75,028	L01EX	Other protein kinase inhibitors
Bosutinib	4.73 (4.51–4.95)	<0.0001	24,978	L01EA	BCR-ABL tyrosine kinase inhibitors
Dacomitinib	4.38 (3.09–6.19)	<0.0001	465	L01EB	EGFR tyrosine kinase inhibitors

HER2, human epidermal growth factor receptor 2; CDK, cyclin-dependent kinase; EGFR, epidermal growth factor receptor; BCR-ABL, breakpoint cluster region-Abelson.

**Table 2:** Cross-tabulation and calculation formula for RORs of diarrhea.

	Diarrhea	Non—Diarrhea
Reports with the suspected medicine	*a*	*c*
All other reports	*b*	*d*

$$ROR_{drug}=\frac{\left(\frac{a_{drug}}{b_{drug}}\right)}{\left(\frac{c_{drug}}{d_{drug}}\right)}$$

### 2.3. Univariate Analysis of Antineoplastic Agents Suspected of Inducing Diarrhea

The principal objective of this study was to characterize diarrhea-inducing drug-specific MIEs detected from the ADE database and to provide toxicological insights. Toxicity Predictor was used to estimate the MIE activity of 155 drugs, excluding 42 drugs due to platinum content or high molecular weight (two drugs) and the missing value of SMILES (40 drugs).

A univariate analysis was performed to identify MIEs associated with diarrhea-inducing drugs from the 155 drugs for which the predicted MIE activity values can be estimated. The diarrhea-inducing drugs were the 45 drugs with lnROR > 0 and −log10[*p*-value] ≥ 1.3 in the volcanic plot. All 56 MIEs that can be predicted using Toxicity Predictor were analyzed (Supplementary Table S1), and the univariate analysis revealed that 28 MIEs were significantly associated with diarrhea-inducing drugs (Table 3).

### 2.4. Multivariate Analysis of Antineoplastic Agents Suspected of Inducing Diarrhea

Owing to a relatively low sample size, the risk of bias may occur in models with a high number of covariates. Therefore, sensitivity analyses were performed using a forward–backward stepwise algorithm to limit the number of covariates, increase model precision, and improve model fit. A *p*-value < 0.1 was used as the criterion for each variable to stay in a model (for backward and stepwise) and to enter a model (for forward and stepwise).

In the multivariate analysis, 28 MIEs found in the univariate analysis to have a significant association with diarrhea-inducing drugs were evaluated. The backward–forward stepwise algorithm selected the final model with one covariate. Progesterone receptor (PR) antagonists were the only drug class found to be significantly associated with diarrhea (0.779, *p* = 0.0002; Table 3).

## 3. Discussion

This study evaluated potential MIEs associated with diarrhea-inducing antineoplastic agents. To the best of our knowledge, this is the first study to investigate the initial interaction between a molecule and a biomolecule or biosystem of diarrhea-inducing antineoplastic agents using the FAERS database, with a focus on MIEs.

Our results showed that protein kinase inhibitors (e.g., neratinib, abemaciclib, afatinib, and lapatinib) are more likely to induce diarrhea than previously reported cell-killing anticancer drugs such as irinotecan and fluoropyrimidine. Protein kinase inhibitor-induced diarrhea is likely multifactorial, and the mechanisms underlying the diarrhea are unclear. One hypothesis is the inhibition of specific kinase targets in the intestinal epithelium. EGFR and vascular endothelial growth factor receptors are highly expressed in the gut, and inhibiting these receptors in the intestine leads to lowered cell proliferation and reduced capillary networks in the intestinal villi [14,15,16,17]. Moreover, EGFR pathways have stimulatory effects on enterocyte proliferation and nutrient and electrolyte transport; inhibiting these pathways causes structural and functional changes [18]. These changes in the intestinal architecture may lead to mucosal atrophy and reduced absorptive capacity of the gut [19]. In this study, MIEs associated with antineoplastic agent-induced diarrhea were investigated using a multivariate analysis with a backward–forward stepwise algorithm. Of the 56 NR and SR pathway agonist/antagonist activities that were evaluated, PR antagonists were found to be significantly associated with diarrhea.

PR is a master regulator in female reproductive tissues that controls developmental processes, proliferation, and differentiation during the reproductive cycle and pregnancy. It is also involved in the progression of endocrine-dependent breast cancer [20]. As a member of the NR family of ligand-dependent transcription factors, its main function is to regulate networks of target gene expression in response to binding its cognate progesterone [21]. It has been reported that PR antagonists inhibit progesterone during pregnancy. Progesterone withdrawal induces the release of inflammatory mediators, including prostaglandins. Prostaglandins cause powerful uterine smooth muscle contractions, leading to the expulsion of fetal or embryonic tissue [22]. PRs are widely distributed in the body and occur in mucosal sites, including the gastrointestinal tract. Recent data from animal models showed that PR expression is increased in patients with constipation [23]. Progesterone may impair colonic smooth muscle contraction through response to receptor G-protein-dependent agonists (e.g., cholecystokinin and acetylcholine) and in response to the direct G-protein activator guanosine-5ction through response to receptor G-protein-dependent agonists (e.g., cholecyssterone is a contributing factor of chronic constipation [24,25,26]. However, the PR antagonist mifepristone, which is commonly used for pregnancy termination, cervical dilatation, and labor induction, was reported to cause diarrhea in 18–43% of patients [27].

In addition, it was reported that PR signaling could occur via mitogen-activated protein kinase activated by epidermal growth factor signaling. The volcano plot in this study showed that several antineoplastic agents that act on the ErbB receptor family (such as EGFR tyrosine kinase inhibitors and human epidermal growth factor receptor 2 tyrosine kinase inhibitors) induce diarrhea. PR antagonists may also be MIEs involved in antineoplastic agent-induced diarrhea [28]. These results could be verified using in vitro or in vivo experiments and be of useful in managing antineoplastic agent-induced diarrhea.

### Limitations

Since FAERS, a spontaneous reporting database, may not provide information on all the patients who were administered drugs, there may be biases in the risks estimated using ROR. This study has several limitations [29,30]. First, mild ADEs are only occasionally reported, whereas severe ADEs are reported more often. This is a known reporting bias, a characteristic of self-reporting databases [31]. Second, FAERS data contain blank cells, and some reports include incorrect characters and numbers; therefore, ADEs and drug names were revised as much as possible in this study. Third, since multiple drugs were administered, it is difficult to determine the exact cause of ADEs [29,30]. This study used information on all drugs, including the “first suspect drugs,” “second suspect drugs,” “concomitant drugs,” and “interactions,” which can lead to many false positives between drugs and ADEs. Therefore, the results should be analyzed by integrating data from other sources, such as SIDER, DailyMed, and the Comparative Toxicogenomics Database (CTD), in the future. Finally, since the MIEs in this study are values predicted using Toxicity Predictor, they are not actual values for the antineoplastic agents. Thus, the MIEs used in this study may only be part of various biochemical pathways associated with diarrhea.

## 4. Materials and Methods

### 4.1. Database Information

Since January 2004, the United States Food and Drug Administration (FDA) has recorded ADEs associated with marketed drugs and therapeutic biological products using the FDA Adverse Event Reporting System (FAERS) database [31]. The FAERS database contains ADE reports that the FDA received from manufacturers as required by regulation and reports received directly from consumers and healthcare professionals. We downloaded the FAERS database and performed our analysis using data reported between April 2004 and September 2020. The analyzed drug was selected from the FEARS database using the Anatomical Therapeutic Chemistry (ATC) classification system recommended by the World Health Organization [32]. We extracted “L01: ANTINEOPLASTIC AGENTS” (222 drugs) from the “ANTINEOPLASTIC AND IMMUNOMODULATING AGENTS” class, which is one of the 14 major ATC classes.

### 4.2. Terminology of ADE

For diarrhea signal detection, we defined the ADE of diarrhea according to the narrow scope of Standardized MedDRA Queries (SMQ version 23.0). To analyze drug-induced diarrhea, we extracted the following seven Preferred Terms (PTs) together as drug-induced diarrhea from the SMQ in MedDRA/J version 23.0 (MedDRA, Herndon, VA, USA): defecation urgency (PT code: 10012110), diarrhea (PT code: 10012735), diarrhea hemorrhagic (PT code: 10012741), diarrhea neonatal (PT code: 10012743), frequent bowel movements (PT code: 10017367), gastrointestinal hypermotility (PT code: 10052402), and postprocedural diarrhea (PT code: 10057585).

### 4.3. Creation of Data Analysis Table

The FAERS data consist of the following seven tables: (a) patient demographic and administrative information (DEMO), (b) drug/biologic information for as many medications as were reported for the ADE (DRUG), (c) MedDRA terms coded for the ADE (REAC), (d) patient outcomes for the ADE (OUTC), (e) report sources for the ADE (RPSR), (f) drug therapy start and end dates for the reported drug (THER), and (g) MedDRA terms coded for the indications for use (diagnoses) for the reported drugs (INDI).

We created a table that integrated data from DRUG and REAC. From DRUG, we extracted drug information regarding the role in the ADE reported as “primary suspected drug,” “secondary suspected drug,” “concomitant,” or “interacting.” Duplicate data were removed or merged based on the primary ID.

### 4.4. Reporting Odds Ratio (ROR) Calculations

We analyzed diarrhea-related drug signals for the pre-processed FAERS data table (Figure 2). In the signal analysis, the diarrhea-related drug reporting rate was assessed using ROR, which is used in pharmacovigilance to detect ADE signals based on equations and 2 × 2 contingency tables (Table 2). We created a 2 × 2 contingency table of drugs and the associated ADEs based on information from the data analysis table (Table 2). The 2 × 2 contingency table cannot be calculated with zero cells, and the estimation is unstable when the cell frequency is small. Therefore, as a correction, 0.5 was added to all cells (Haldane Anscombe half correction) [33,34]. In this study, antineoplastic agents associated with diarrhea were defined as drugs with ROR ≥ 1 and Fisher’s exact test *p*-value < 0.05 [35]. The threshold for the lower limit was defined according to the value of (*a* + *c*) in Table 2, i.e., the total number of reports on the use of a drug for a specific indication. A threshold value of 100 f reports on the use of a drug for a specific indication. We extracted only reliable RORs by setting this threshold.

### 4.5. Construction of Scatter Plot

We created a scatter plot of ROR and *p*-value for visual interpretation of the ADEs for 197 drugs reported as (*a* + *c*) ≥ 100. The scatter plot was created by converting ROR to positive logarithmically transformed ROR (lnROR) and converting the *p*-value obtained from Fisher’s exact test to logarithmically transformed inverse *p*-value (−log10[*p*-value]). The scatter plot corresponds to volcano plots commonly used in bioinformatics to understand gene expression trends [36,37,38,39,40].

### 4.6. MIE Activity Prediction Using QSAR Toxicity Predictor

We used Toxicity Predictor [41], our previously developed QSAR platform based on machine learning models trained on the Tox21 10 K compounds library, to evaluate agonist and antagonist activity against a total of 56 MIEs. Using Toxicity Predictor, the simplified molecular-input line-entry system (SMILES) strings were cleaned and standardized (by removing salts, counterions, and fragments, and adjusting the protonation state [neutralized]), optimal three-dimensional conformers were determined, and MIE activity predictions were conducted based on molecular descriptors calculated from the optimal conformer. The optimized molecular structures were confirmed using MarvinView (ChemAxon Kft., Budapest, Hungary). We calculated the agonist and antagonist activity values (MIE activity) of NR and SR pathways for each of the 155 compounds, selecting 56 MIEs with an area under the curves ≥0.7, which indicates predictive performance in Toxicity Predictor. The 3 MIEs were excluded from this analysis because calculating the actual values of antineoplastic agents was difficult. Cutoff values for predicted MIE activity were calculated using the Youden method [42], and the calculated MIE activities were normalized to obtain a cut-off value of 0.5. Therefore, predicted labels of compounds with values >0.5 as normalized predicted values were converted to 1, and those with predicted values <0.5 were converted to 0 (Supplementary Table S2).

### 4.7. Multivariate Analysis

Multivariate logistic regression analysis was used to evaluate MIEs associated with diarrhea. Univariate analysis was first performed to extract MIEs that showed a significant positive correlation with ROR of diarrhea using a response variable defined as discrimination between an antineoplastic agent with −log10[*p*-value] ≥ 1.3 and lnROR > 0 and other antineoplastic agents.

For multivariate analysis, we adopted MIEs that showed a significant correlation (*p* < 0.05) with the objective variable in univariate analysis. The variables adopted for multivariate analysis were screened for multicollinearity (Pearson’s correlation coefficient |R| > 0.9). A multivariate ordered logistic regression model was constructed using a stepwise forward selection method with a variable entry criterion of 0.10 and a variable retention criterion of 0.10. The a priori level of significance was set at 0.05.

### 4.8. Statistical Analysis

All analyses were performed using JMP Pro 14 (SAS Institute Inc., Cary, NC, USA), and the level of statistical significance was set to 0.05.

## 5. Conclusions

By analyzing a large dataset, we identified the potential MIEs associated with diarrhea-inducing antineoplastic agents. Our analysis revealed two important findings. First, protein kinase inhibitors are more likely to induce diarrhea than the previously reported diarrhea-inducing cell-killing anticancer drugs, such as irinotecan and fluoropyrimidine. Second, progesterone receptor antagonists may be potential MIEs associated with diarrhea. These findings may facilitate the prediction and management of antineoplastic agent-induced diarrhea.

## Figures and Tables

**Table 3:** Univariate analysis and multivariate analysis of MIEs associated with diarrhea-inducing drugs.

Variable	Univariate Analysis				Multivariate Analysis	
	ROR	95% CI		*p*-Value	ROR	*p*-Value
		Lower	Upper			
GR antagonist	5.342	1.54	18.59	0.0024	—	—
PR antagonist	4.750	2.08	10.84	<0.0001	0.779	0.0002
VDR agonist	4.537	1.65	12.47	0.0011	—	—
TGFb antagonist	4.071	1.67	9.95	0.0008	—	—
ERb antagonist	3.919	1.60	9.58	0.0012	—	—
ARfull antagonist	3.831	1.39	10.58	0.0043	—	—
ARfulls antagonist	3.619	1.48	8.86	0.0023	—	—
ERR agonist	3.619	1.48	8.86	0.0023	—	—
Shh antagonist	3.537	1.37	9.13	0.0045	—	—
PPARd agonist	3.273	1.50	7.14	0.0017	—	—
PPARg antagonist	3.250	1.26	8.41	0.0082	—	—
PPARg agonist	3.119	1.37	7.12	0.0040	—	—
Arom antagonist	3.113	1.20	8.06	0.0111	—	—
ERlbd antagonist	3.083	1.31	7.27	0.0058	—	—
ERR agonist	3.079	1.25	7.57	0.0082	—	—
PPARd antagonist	2.885	1.26	6.60	0.0074	—	—
ARlbd agonist	2.714	1.10	6.69	0.0190	—	—
PXR agonist	2.565	1.24	5.32	0.0082	—	—

GR, glucocorticoid receptor; PR, progesterone receptor; VDR, vitamin D receptor; TGFb, transforming growth factor beta; ERb, estrogen receptor beta; ARfull, androgen receptor full antagonist; ARfulls, androgen receptor with stimulator; ERR, estrogen-related receptor; Shh, sonic hedgehog signaling; PPARd, peroxisome proliferator-activated receptor delta; PPARg, peroxisome proliferator-activated receptor gamma; Arom, aromatase; ERlbd, estrogen receptor alpha lbd; ARlbd, androgen receptor lbd; RXR, retinoid X receptor alpha.

## Data Availability

Data is contained within the article.

## Supplementary Materials

The following supporting information can be downloaded at: https://www.mdpi.com/article/10.3390/ijms232012407/s1.
