# Supplementary material for: Nuclear Receptor and Stress Response Pathways Associated with Antineoplastic Agent-Induced Diarrhea
Source: Int J Mol Sci. 2022 Oct 17;23(20):12407. doi: 10.3390/ijms232012407 (PMC9604027; doi:10.3390/ijms232012407)
Supplement: Supplementary file 1 [file ijms-23-12407-s001.zip › Supplementary Table S1.pdf]

Supplementary Table S1. Univariate analysis of MIEs associated with diarrhea-inducing drugs

Variable	ROR	Univariate analysis		*P*-value
		95% CI		
		Lower	Upper	
PR antagonist	4.750	2.08	10.84	<.0001
TGFb antagonist	4.071	1.67	9.95	0.001
VDR agonist	4.537	1.65	12.47	0.001
ERb antagonist	3.916	1.60	9.58	0.001
PPARd agonist	3.273	1.50	7.14	0.002
Arfulls antagonist	3.619	1.48	8.86	0.002
ERR agonist	3.619	1.48	8.86	0.002
GR antagonist	5.342	1.54	18.59	0.002
PPARg agonist	3.119	1.37	7.12	0.004
ARfull antagonist	3.831	1.39	10.58	0.004
Shh antagonist	3.537	1.37	9.13	0.005
ERlbd antagonist	3.083	1.31	7.27	0.006
PPARd antagonist	2.885	1.26	6.60	0.007
ERR antagonist	3.079	1.25	7.57	0.008
RXR agonist	2.565	1.24	5.32	0.008
PPARg antagonist	3.250	1.26	8.41	0.008
Arom antagonist	3.113	1.20	8.06	0.011
ARlbd antagonist	2.714	1.10	6.69	0.019
PXR agonist	2.325	1.10	4.92	0.019
MMP disruptor	2.257	1.08	4.72	0.022
FXR agonist	2.196	1.07	4.49	0.023
TSHR antagonist	2.113	1.03	4.32	0.029
ARlbd agonist	2.154	1.02	4.56	0.031
ATAD5 inducer	2.071	1.01	4.26	0.034
Erfulls antagonist	2.144	0.98	4.69	0.039
ARE agonist	2.380	0.96	5.89	0.041
VDR antagonist	2.154	0.96	4.82	0.043
FXR antagonist	1.985	0.89	4.45	0.066
GR agonist	1.865	0.89	3.90	0.068
TR antagonist	2.000	0.87	4.61	0.072
ERRPGC agonist	2.000	0.87	4.61	0.072

CaspC inducer	1.778	0.87	3.65	0.081
ERfull antagonist	1.776	0.84	3.77	0.092
CAR antagonist	1.692	0.78	3.65	0.122
ERRPGC antagonist	1.621	0.78	3.36	0.131
HSR activator	1.727	0.77	3.87	0.132
CaspH inducer	1.524	0.76	3.07	0.158
AhR agonist	1.417	0.70	2.88	0.216
ERsr agonist	1.288	0.63	2.63	0.302
p53 agonist	1.295	0.58	2.88	0.335
HIF1 agonist	1.213	0.60	2.45	0.359
TRHR agonist	1.214	0.52	2.86	0.405
ERlbd agonist	1.153	0.57	2.33	0.413
ERaant agonist	1.429	0.33	6.25	0.449
NFkB agonist	1.132	0.47	2.74	0.474
Shh agonist	1.089	0.39	3.07	0.532
TRHR antagonist	0.848	0.40	1.82	0.728
TSHR agonist	0.809	0.36	1.80	0.762
CAR agonist	0.800	0.34	1.89	0.763
HDAC antagonist	0.695	0.24	2.03	0.821
H2AX agonist	0.745	0.37	1.51	0.841
TGFb agonist	0.655	0.28	1.53	0.884
ARant agonist	0.216	0.03	1.74	0.983
PR agonist	0.216	0.03	1.74	0.983
ERb agonist	0.403	0.15	1.05	0.985
AP1 agonist	0.464	0.22	0.98	0.987

PR, progesterone receptor; TGFb, transforming growth factor beta; VDR, vitamin D receptor; ERb, estrogen receptor beta; PPARd, peroxisome proliferator-activated receptor delta; Arfulls, androgen receptor with stimulator; ERR, estrogen related receptor; GR, glucocorticoid receptor; PPARg, peroxisome proliferator-activated receptor gamma; ARfull, ; Shh, sonic hedgehog signaling; ERlbd, estrogen receptor alpha lbd; RXR, retinoid X receptor-alpha; Arom, aromatase; ARlbd, androgen receptor lbd; PXR, human pregnane X receptor; MMP, mitochondrial membrane potential; FXR, farnesoid-X-receptor; TSHR, thyroid stimulating hormone receptor; ATAD5, ATAD5 genotoxic; Erfulls, estrogen receptor alpha with stimulator; ARE, antioxidant response element; TR, thyroid receptor; ERRPGC, estrogen related receptor with PGC; CaspC, caspase-3/7 in CHO-K1; ERfull, estrogen receptor alpha full; CAR, constitutive androstane receptor; HDAC, histone deacetylase; H2AX, histone variant H2AX; ARant, androgen receptor with antagonist; AP1, activator protein-1
